# Personalized Sentinel Node Mapping in Endometrial Cancer by the Indocyanine Green Implementation as Single Tracer: A Case Control Study

**DOI:** 10.3390/jpm13020170

**Published:** 2023-01-18

**Authors:** Ignacio Cristóbal Quevedo, Ignacio Cristóbal García, Myriam Gracia, Virginia Garcia-Pineda, Maria Alonso-Espias, Jaime Siegrist, Maria Dolores Diestro, Alicia Hernández, Ignacio Zapardiel

**Affiliations:** 1 Gynecologic Oncology Unit, La Paz University Hospital, 28046 Madrid, Spain; 2 Gynecology Department, San Carlos Clinic Hospital, 28040 Madrid, Spain; 3 Instituto de Investigación Sanitaria Hospital Clínico San Carlos de Madrid (IdISSC), 28040 Madrid, Spain

**Keywords:** endometrial neoplasms, sentinel lymph node, indocyanine green, technetium

## Abstract

The main objective was to analyze the rate of bilateral sentinel lymph node (SLN) detection in endometrial cancer using indocyanine green (ICG) as a unique tracer compared to Technetium99 + ICG. As secondary objectives, we analyzed the drainage pattern and factors that might affect the oncological outcomes. A case-control ambispective study was carried out on consecutive patients at our center. Data on the SLN biopsy with ICG collected prospectively were compared to retrospective data on the use of a double-tracer technique including Technetium99 + ICG. In total, 194 patients were enrolled and assigned to both groups, in which the group with both tracers (controls) included 107 (54.9%) patients and the ICG-alone group (cases) included 87 (45.1%) patients. The rate of bilateral drainage was significantly higher in the ICG group (98.9% vs. 89.7%; *p* = 0.013). The median number of nodes retrieved was higher in the control group (three vs. two nodes; *p* < 0.01). We did not find survival differences associated with the tracer used (*p* = 0.85). We showed significant differences in terms of disease-free survival regarding the SLN location (*p* < 0.01), and obturator fossa retrieved nodes showed better prognosis compared to external iliac. The use of ICG as a single tracer for SLN detection in endometrial cancer patients seemed to obtain higher rates of bilateral detection with similar oncological outcomes.

## 1. Introduction

Currently, endometrial cancer represents the sixth most frequent cancer in the world, being the second most frequent of gynecological origin in Europe [1] and eighth in terms of causing deaths in Europe [2]. It is estimated that 320,000 new cases of endometrial cancer are diagnosed each year, presenting an approximate 5-year survival rate of 76% [3,4].

Nodal status is one of the most important prognostic factors, allowing better adjuvant treatment planning. The need to systematically perform lymphadenectomies in early-stage tumors is a matter of controversy as it may not provide therapeutic benefits [5,6,7], increasing morbidity such as lymphedema, or increased blood loss and surgical time [8]. Sentinel lymph node (SLN) mapping has shown false negative rates of 2.8% [9] and 4.3% when selecting only high-risk tumors [10], and it is replacing full-nodal dissection in the current practice.

Different tracers have been used to detect SLN in endometrial cancer. The injection of Technetium99 (Tc99m) radiocolloid in the cervix and its detection in the operating room is one of the original forms of SLN mapping [11,12], which results in an overall detection rate of approximately 90% and a bilateral detection rate of 60%, commonly used in combination with other tracers [13,14,15,16,17].

Indocyanine green (ICG) has been progressively introduced as a tracer [18]. It allows real-time mapping using near-infrared images intraoperatively [19]. A meta-analysis demonstrated that indocyanine green SLN detection appears to be at least equivalent to the combination of blue dye and Tc99m in terms of overall and bilateral detection rates [20]. 

In the FILM study, 471 (97%) of 485 lymph nodes were identified with ICG and 226 (47%) with blue dye (difference 50%, CI95% 39–62%; *p* < 0.0001), presenting how ICG is superior to the blue dye [21]. Another study that compared ICG vs. blue dye, one tracer in each hemipelvis, proved that the use of ICG instead of blue dye results in a 26.5% (CI95% 17.4–35.6%) increase in SLN detection rates per hemipelvis in women with endometrial cancer [22].

In fact, ICG seems to be the most accurate tracer in early-stage endometrial cancer for SLN mapping, showing overall detection rates of 95% and bilateral detection rates of 80% [9,23,24].

In other studies, where they compared the use of Tc99m and blue dye vs. ICG with only ICG, the overall detection rate of SLN biopsy was 97.3% (143/147) for women in the double-tracer group and 96.9% (189/195) for women in the ICG group (*p* = 0.547), although the bilateral rate for ICG was 84.1%, significantly higher with respect to the 73.5% obtained with the double-tracer group (*p* = 0.007) [5]. Buda et al. [25], compared blue dye and Tc99m vs. ICG, obtaining detection rates of SLN mapping of 96% and 100% for Tc99m + blue dye and ICG, respectively; bilateral mapping was achieved in 98.5% for ICG and 76.3% for Tc99m + blue dye with a significant difference (*p* < 0.0001).

Few papers have been published comparing ICG with Tc99m + ICG, but in a study [26] that compared Tc99m alone vs. ICG + Tc99m, the total bilateral detection rate was 72.1%, without significant differences between ICG as a single tracer and Tc99m + ICG (75% vs. 87.3%, respectively; *p* = 0.1). In a recent meta-analysis [27], researchers showed that SLN mapping with ICG has a high overall detection rate of 95.6% (95% CI = 92.4–97.9%) and a bilateral detection rate of 76.5% (95% CI = 68.1–84.0) in low- and intermediate-risk endometrial cancer patients.

The use of ICG may decrease the overall number of complete lymphadenectomies [28], thus impacting the quality of life and morbidity of the patients without impairing the oncological outcomes supporting the validity of ICG nodal mapping [29].

The main objective of this study was to analyze the rate of bilateral SLN detection in endometrial cancer by using ICG alone. As secondary objectives, we analyzed the drainage pattern and perioperative factors that could affect the detection of SLN and the survival of patients.

## 2. Materials and Methods

We conducted a case-control study collecting data prospectively from all consecutive patients diagnosed with endometrial cancer and operated on between 15 February 2019 and 31 December 2021 in which SLN detection was performed using ICG alone (Group 2). They were compared with a retrospective control group of patients operated on consecutively from 1 January 2016 to 14 February 2019 in which the double-tracer technique using both ICG and Tc99m radiocolloid was used for SLN detection (Group 1).

The study was approved by the Ethics Committee of University Hospital La Paz (Ref. #PI-3599). All women eligible for the study signed a written informed consent at the time they were scheduled for surgery. The retrospective comparison group also had previously informed consent for the surgery.

The inclusion criteria were patients older than 18 years; the absence of iodine allergy; pathologic confirmation of endometrial cancer; preoperative FIGO stage I-III; no medical or anesthetic contraindication for surgery; and acceptance of the Informed Consent. In our study, we also included patients with atypical hyperplasia suspicious of carcinoma as high-risk histology types, as outlined in the ESGO guidelines. Among the exclusion criteria, we established preoperative FIGO stage IV; pregnancy; a history of pelvic and/or abdominal irradiation; and patients who did not meet all the inclusion criteria.

The same gynecologic oncology team, considering the following technical aspects, carried out all surgical procedures. For the Tc99m + ICG patients, the day before surgery, two cervical injections (5 mm superficial and 15 mm deep) of 2 mL of technetium sulfur colloid were administered at 3 and 9 o’clock. Lymphoscintigraphy images were obtained 2 h after the injections with the integration of single-photon emission computed tomography (SPECT/CT). The indocyanine green injection was performed 10 min before the detection of the pelvic SLN and always after performing para-aortic lymphadenectomy if it was indicated. Twenty-five milligrams of ICG were diluted in 10 cc of distilled water and injected 1 cc deeply and superficially into the cervix at 3 and 9 o’clock (4 cc in total). A near-infrared camera (Olympus Medical Systems Europa, Hamburg, Germany, or Stryker Iberia S.L., Madrid, Spain) was used in overlay mode. The first node of the lymphatic chain identified by ICG and the accessory nodes that raise doubts or seemed infiltrated were removed. Patients with high-risk endometrial cancer who underwent surgery prior to 2019 also underwent pelvic and para-aortic lymphadenectomy as part of the past protocol of our center.

All SLNs were analyzed postoperatively through the ultrastaging method. The adjuvant treatment was indicated in the Multidisciplinary Gynecologic Oncology tumor board for all patients. Follow-up frequency was carried out following the protocols of the Gynecology Oncology Unit with a maximum follow-up period of ten years.

### Statistical Analysis

Quantitative variables were described using the mean and standard deviation, and qualitative variables by absolute values and proportions. Quantitative variables between the two groups were compared using Student’s *t*-test or an analysis of variance and the Bonferroni post-hoc for groups with more than two categories. Qualitative variables between groups were analyzed with the Chi-square test. The univariate and multivariate analysis was carried out by means of logistic regression. The Kaplan–Meier curve and the log-rank test were used to evaluate the survival. All statistical analyses were performed using the statistical package SPSS.17.0 (SPSS Inc., Madrid, Spain). The α-error was set at 5%, and all statistical comparisons were two-sided.

## 3. Results

A total of 194 patients were enrolled and assigned to both groups. Group 1 included 107 (54.9%) patients and Group 2 had 87 (45.1%) patients. Laparoscopic surgery was performed in 191 patients (97.9%). The demographic and presurgical clinical data of both groups are summarized in Table 1.

Surgical procedures and pathological results are shown in Table 2. No significant differences were observed in the SLN locations between both groups, with the obturator fossa being the most frequent location in both groups. A significant difference in the bilateral detection rate was observed between group 1 and group 2 (89.7% vs. 98.9%, respectively; *p* = 0.013). A higher median number of SLNs was intraoperatively detected in group 1 compared to group 2 (3 vs. 2, respectively; *p* < 0.01) and confirmed in the final pathology (*p* = 0.02). However, no differences between groups were observed in terms of metastatic nodal detection (*p* = 0.15) or most pathologic characteristics, as well as the final FIGO stage.

Follow-up and adjuvant treatment is presented in Table 3. Significant differences were observed in terms of cancer relapse. The relapse rate in group 1 was 19.6% compared to 6.9% in group 2 patients (*p* = 0.012). In addition, statistical differences (*p* < 0.01) were observed in terms of the death rate, as in group 1, it was 14.3% compared to 1.2% in group 2. However, after multivariable analysis, no significant differences were observed between groups in terms of possible associated risk factors and FIGO stage (*p* = 0.128). We also did not find survival differences associated with the tracer used (*p* = 0.85) or the number of SLN detected intraoperatively (*p* = 0.6). Lymphedema occurred in eight (7.5%) of our group 1 patients and four (4.6%) of the second group, without significant differences (*p* = 0.551).

**Figure 1:**
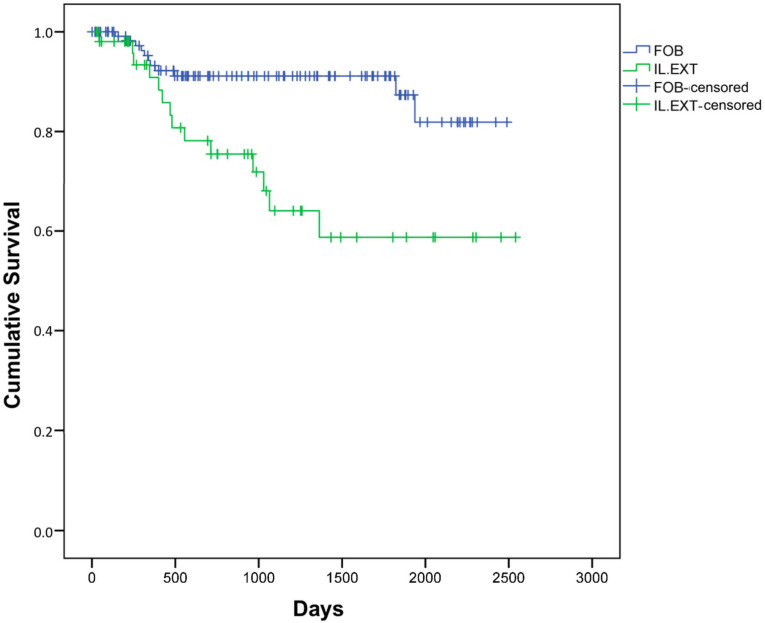
Kaplan–Meier estimate. Disease-free survival comparing sentinel node location (FOB: Obturator Fossa; IL. EXT: External Iliac Artery). Log-rank test *p* < 0.01.

Interestingly, we showed significant differences in terms of disease-free survival regarding the SLN location (*p* < 0.01), as patients with the obturator fossa SLN anatomic location showed better prognosis compared to those with external iliac nodes (Figure 1). No differences were observed in the overall and disease-free survival rates in the Kaplan–Meier estimate between both groups in any of the studied variables.

## 4. Discussion

Our study showed that the use of ICG alone in the detection of sentinel node biopsy for endometrial cancer has a better migration pattern than the combination of Tc99m and ICG in terms of the bilateral detection rate and a lower nodal retrieval. In addition, no significant differences between both groups were observed in disease-free and overall survival rates when comparing the tracer technique used, although differences were observed in terms of the SLN anatomical location, as patients with SLN retrieved at the obturator fossa had better disease-free survival than the iliac external node location.

The FILM study [21], a randomized controlled non-inferiority trial that compared blue dye and ICG, concluded that infrared-based fluorescence techniques such as ICG showed a higher detection rate of sentinel lymph nodes compared to other options. In this study, bilateral sentinel nodes were identified in 134 (82%) patients, 52 (32%) with blue dye and 132 (81%) with indocyanine green (*p* < 0.0001) [21]. Comparing this with our study, we showed 90% with the double-tracer protocol and nearly 99% bilateral SLN detection with ICG alone, improving the numbers of bilateral rates in the FILM study. Furthermore, another randomized controlled trial highlighted the use of ICG instead of blue dye, resulting in a significant increase in SLN detection rates per hemipelvis [22], although when describing the bilateral rates, this cannot be considered relevant as no single dye was injected bilaterally in the same patient. However, they achieved an overall detection rate with ICG of 95.5% (126/132) reflecting the high mapping rate with ICG, with similar numbers to ours, as we had a detection rate of 96.9% with ICG alone. A previous meta-analysis [31,32] studied the use of ICG and demonstrated a significant improvement in the overall SLN detection rate compared to Tc99m as ICG use improved overall (94%, CI95% 92–96%, 19 studies) SLN detection rates compared to the blue tracer (86%, CI95% 83–89%, 31 studies) or Tc99m (86%, CI95% 83–89%, 25 studies). We had better detection rates with ICG, though our detection rates with the double tracer were lower than the one published in the meta-analysis; this trend was similarly seen in terms of bilateral detection rates (74% vs. 59% vs. 57%, respectively), although not achieving our bilateral rates or significance. In addition, a study [26] that compared Tc99m alone vs. ICG + Tc99m, with a lower number of patients, had a non-significant better bilateral rate with the combination of both tracers than ICG alone (87.3% vs. 75%). In comparison with our study, we had a significant difference in favor of ICG alone and higher percentages of detection of approximately 99% and 90%, respectively. It has been suggested that ICG provides better tissue penetration than the other tracers used, as it may be suggested by our findings, and also makes identification easier for the surgeon [33,34]. In addition, comparing our results with a recent meta-analysis [27] of patients with low- and intermediate-risk endometrial cancer that used ICG as an SLN tracer, we achieved a higher bilateral rate of SLN mapping with ICG with similar overall detection rates, proving the ICG is a more suitable tracer option. Although this meta-analysis only included the ICG data of the studies included, several of them used multiple tracers and small populations of patients for ICG were used.

We observed a significantly higher number of node retrievals among the group using combined tracers. Some authors [35] explain this finding as an expected higher sensitivity for the combined technique compared to ICG alone (100% vs. 92.5%, respectively). However, in our study, this aspect was not demonstrated. In fact, the percentage of women with positive nodes missed does not differ according to the tracer used. What has been shown is that at the initial stages of ICG, using a learning curve, a higher number of SLNs are identified, which decreases with the surgeon’s experience, being reduced to the minimum over years of experience of our center, with the use of IC, currently being more precise [36]. Our median SLN node number seen intraoperatively with ICG was 2 (2–3) compared to 3 (2–4) using the double-tracer technique (*p* = 0.006). A similar number of nodes was retrieved in the FILM study using ICG, as the mean number of nodes per patient was 3.1 nodes [SD 1.7], finding ICG to be superior to blue dye. Other studies had a median number of 4 (range 1–7) nodes [37], being superior to the ones we noted. The higher numbers of SLNs detected intraoperatively were confirmed histologically, although no differences were observed according to the time, likely because we implemented the technique 10 years ago and the learning curve was already completed for all patients included in the present study. We think that the combined use of both tracers could cause Tc99m to act as a confounding factor since both tracers share the same lymphatic channels and could affect the distribution of ICG (which is always injected after Tc99m). In our opinion, by using only ICG, the surgeon can focus more on the anatomic localization and distribution of the lymphatic channels and nodes, resulting in a more precise dissection and nodal retrieval.

We did not find differences in the number of patients with positive SLN nodes (*p* = 0.094), nor in their pathology, perhaps caused by the use of ICG as the complementary tracer to Tc99m. Studies [21] have concluded that all metastatic sentinel nodes were detected with ICG, but more than one-third would have been missed if blue dye alone would have been used, as 16 (9%) of 176 patients had metastatic disease in 21 sentinel nodes, and among them, 13 (62%) were positive for both blue and green and 8 (38%) were positive for green only.

We observed significantly worse oncological results in the descriptive study among the group of patients with combined tracers, but this finding could be explained by a statistically higher lymphovascular space invasion and higher FIGO stage in that group, though this last item was not statistically significant due to the larger number of days of follow-up in the double-tracer group. It is known that lymphovascular space invasion is an independent prognostic impact regardless of the genetic subtype, increasing the risk of death of any cause, death due to endometrial carcinoma, and recurrent or progressive disease by 1.5–2 times [38]. No other significant risk factors were observed in the multivariate test performed when comparing the oncological results.

No differences have been observed in the Kaplan–Meier estimate between both groups of the tracers in terms of disease-free and overall survival, and the number of SLNs seen during surgery is not associated with disease-free and overall survival rates. Although, currently, several studies have compared types of tracers for SLN on endometrial cancer, no studies have been found describing the survival comparison depending on the tracer used, but we may extrapolate that the better the tracer used, the more precise we are with the SLN retrieval and the better the survival rates. No differences have been observed in the overall survival rate in terms of the anatomical localization of the SLN. However, differences in the descriptive analysis, as shown in Table 3, were observed in terms of disease-free or overall survival and total deaths. This can be explained by the fact that we had longer follow-ups in the double-tracer group than in the ICG group.

Differences have been observed in disease-free survival in terms of the SLN localization in the Kaplan–Meier estimate analysis, as patients with obturator fossa nodes had a higher rate than the ones with exterior iliac artery SLNs localization. Presenting SLNs at the obturator fossa reduces the risk of relapse by 69.8%. As is known [29,31], approximately 5% of SLNs can jump to other less common areas, areas not routinely dissected in pelvic lymphadenectomies. It is reasonable to say that in higher-level SLN, more probabilities of unusual drainage can appear, and metastasis in a lymphatic node is more likely, worsening oncological outcomes [39]. One study [29] described the SLN locations as follows: External iliac region (71%), internal iliac (2%), obturator fossa (10%), common iliac (12%), para-aortic (2%), sacral (2%), and parametrial (1%); however, our most frequent location was the obturator fossa and the external iliac artery ranked second, as shown in Table 2, although no differences were observed in the location between the different tracers used.

The main strength of this study is the assessment of the bilateral detection rate using ICG as a single tracer in a prospective nature with sufficient sample size, though a short follow-up period was performed in this group as part of a preliminary study. In contrast, the main weakness is that the control group data were collected retrospectively, which includes some possible bias due to the lack of accurate information. In addition, the study was conducted in a single center with more than 10 years of experience in SLN biopsy, which may not be applicable to all centers. However, this study opens the door for the application of ICG alone for the surgical treatment of endometrial cancer, reducing the number of full lymphadenectomies performed and avoiding the use of nuclear medicine to perform the SLN biopsy.

## 5. Conclusions

The use of ICG as a single tracer in the detection of sentinel node biopsy for endometrial cancer in this initial study seems to offer a better detection rate than the combination of ICG and Tc99m in terms of bilateral migration, additionally showing a lower but more accurate nodal retrieval. In addition, no significant differences between both groups were observed in disease-free and overall survival rates when comparing the tracer technique used.

## Figures and Tables

**Table 1:** Demographic and clinical data of both groups (*n* = total number of patients; SD = standard deviation; ICG = indocyanine green; Tc99m = technetium99; MRI = Magnetic resonance imaging; US = ultrasound; * Significant differences).

Characteristics		Group 1 (Tc99m + ICG) *n* = 107	Group 2 (ICG) *n* = 87	*p* Value
**Age (mean, SD)**		63.07 ± 11.39	62.06 ± 10.88	0.532
**Body mass index (median, range)**		28.8 (24.95–32)	28.3 (25.75–34.1)	0.451
**Hypertension (*n*,%)**		38 (35.5%)	35 (40.2%)	0.552
**Diabetes (*n*, %)**		21 (19.6%)	7 (8%)	0.025 *
**Premenopausal (*n*,%)**		11 (10.3%)	9 (10.3%)	1.0
**Previous uterine disorders**				>0.05
	Myomectomy/C-Section (*n*,%)	1 (0.9%)	7 (8%)	
	Uterine Fibroids (*n*,%)	22 (20.6%)	9 (10.3%)	
	Adenomyosis (*n*,%)	1 (0.9%)	1 (1.1%)	
**Presurgical Pathology Biopsy**				0.181
	Endometroid adenocarcinoma (*n*,%)	90 (84.1%)	79 (90.8%)	
	Clear Cell carcinoma (*n*,%)	5 (4.7%)	3 (3.4%)	
	Serous carcinoma (*n*,%)	9 (8.4%)	3 (3.4%)	
	Carcinosarcoma (*n*,%)	1 (0.9%)	1 (1.1%)	
	Atypical hyperplasia (*n*,%)	1 (0.9%)	1 (1.1%)	
	Neuroendocrine carcinoma (*n*,%)	1 (0.9%)	0	
**Tumor Grade**				0.002 *
	Grade 1 (*n*,%)	54 (50.5%)	64 (73.6%)	
	Grade 2 (*n*,%)	33 (31.1%)	10 (11.6%)	
	Grade 3 (*n*,%)	19 (17.9%)	12 (14%)	
**MRI (presurgical FIGO stage)**				>0.05
	IA (*n*,%)	45 (42.1%)	18 (20.7%)	
	IB (*n*,%)	33 (30.8%)	17 (19.5%)	
	II, IIIA/B (*n*,%)	4 (3.7%)	9 (10.3%)	
**US (presurgical FIGO stage)**				0.634
	IA (*n*,%)	48 (44.9%)	41 (47.1%)	
	IB (*n*,%)	35 (32.7%)	33 (37.9%)	
**FIGO Presurgical Stage**				>0.05
	IAG1 (*n*,%)	40 (37.4%)	42 (48.3%)	
	IAG2 (*n*,%)	18 (16.8%)	3 (3.4%)	
	IAG3 (*n*,%)	11 (10.3%)	6 (6.9%)	
	IBG1 (*n*,%)	13 (12.1%)	18 (20.7%)	
	IBG2 (*n*,%)	13 (12.1%)	5 (5.7%)	
	IBG3 (*n*,%)	8 (7.5%)	4 (4.6%)	
	II (*n*,%)	1 (0.9%)	3 (3.4%)	
	IIIA (*n*,%)	1 (0.9%)	3 (3.4%)	
	IIIB (*n*,%)	1 (0.9%)	0	
	IIIC1 (*n*,%)	1 (0.9%)	3 (3.4%)	

**Table 2:** Surgical procedures of both groups and final pathology results (*n* = total number of patients; SD = standard deviation; ICG = indocyanine green; Tc99m = technetium99; HTDA = hysterectomy and double adnexectomy; SLN = sentinel lymph node; LP = pelvic lymphadenectomy; LPAO = paraaortic lymphadenectomy; * Significant differences).

Variables			Group 1 (Tc99m + ICG) *n* = 107	Group 2 (ICG) *n* = 87	*p* Value
**Surgical procedures**					>0.05
	HTDA + SLN (*n*,%)		62 (57.9%)	72 (82.8%)	
	HTDA + SLN + LP + LPAO (*n*,%)		29 (27.1%)	7 (8%)	
	HTA + SLN + LP + LPAO + omentectomy (*n*,%)		11 (10.3%)	7 (8%)	
	HTDA + SLN + LP (*n*,%)		4 (3.7%)	1 (1.1%)	
	HTDA + LP (*n*,%)		1 (0.9%)	0	
**Retroperitoneal lymphadenectomy (*n*,%)**			26 (24.3%)	8 (9.2%)	0.535
**Laparoscopy (*n*,%)**			104 (97.2%)	86 (98.6%)	>0.05
**Laparotomy (*n*,%)**			3 (2.8%)	1 (1.1%)	>0.05
**Tracer used**					>0.05
	ICG + Tc99m (*n*,%)		82 (76.6%)	0	
	ICG (*n*,%)		20 (18.7%)	84 (96.6%)	
	Tc99m (*n*,%)		5 (4.7%)	0	
**Bilateral Sentinel Node (*n*,%)**			96 (89.7%)	87 (98.9%)	0.013 *
**Sentinel Node Location**					>0.05
	Obturator Fossa (*n*,%)		74 (69.2%)	61 (70.1%)	
	Exterior Iliac Artery (*n*,%)		61 (57%)	44 (50.6%)	
	Common Iliac Artery (*n*,%)		22 (20.6%)	5 (5.7%)	
	Hypogastric Artery (*n*,%)		1 (0.9%)	7 (8%)	
	Paraaortic (*n*,%)		1 (0.9%)	0	
	Presacral (*n*,%)		2 (1.2%)	1 (1.1%)	
**Number of Nodes observed intraoperatively**					
	Sentinel Node (median, range)		3 (2–4)	2 (2–3)	0.006 *
	LP (mean, SD)		13.74 ± 5.39	8.72 ± 7.04	0.003 *
	LPAO (mean, SD)		16.49 ± 8.06	7.81 ± 7.52	0.001 *
**Final Pathology**					
Sentinel Node number (median, range)			3 (2–4)	2 (2–3)	0.023 *
Sentinel Node Pathology					0.150
		Negative (*n*,%)	92 (86%)	82 (94.3%)	
		Isolated tumoral cells (*n*,%)	5 (4.7%)	1 (1.1%)	
		Micrometastasis (*n*,%)	3 (2.8%)	0	
		Macrometastasis (*n*,%)	6 (5.6%)	4 (4.6%)	
Patients with positive nodes (*n*,%)			14 (13.2%)	5 (5.75%)	0.094
Pelvic Lymphadenectomy					>0.05
		Negative (*n*,%)	42 (39.3%)	12 (13.8%)	
		Micrometastasis (*n*,%)	1 (0.9%)	0	
		Macrometastasis (*n*,%)	3 (2.8%)	2 (2.3%)	
Paraaortic lymphadenectomy					>0.05
		Negative (*n*,%)	38 (35.5%)	10 (11.5%)	
		Macrometastasis (*n*,%)	3 (2.8%)	2 (2.3%)	
Myometrial Invasion					>0.05
		<50% (*n*,%)	67 (62.6%)	69 (79.3%)	
		>50% (*n*,%)	39 (37.4%)	18 (20.6%)	
Histology					0.128
		Endometroid (*n*,%)	91 (85%)	78 (89.7%)	
		Clear Cell (*n*,%)	3 (2.8%)	2 (2.3%)	
		Serous (*n*,%)	9 (8.4%)	4 (4.6%)	
		Carcinosarcoma (*n*,%)	4 (3.7%)	1 (1.1%)	
		Atypical Hyperplasia (*n*,%)	0	2 (2.3%)	
Tumor Grade					0.117
		Grade 1 (*n*,%)	57 (53.3%)	59 (67.8%)	
		Grade 2 (*n*,%)	27 (25.2%)	16 (18.4%)	
		Grade 3 (*n*,%)	23 (21.5%)	12 (13.8%)	
Lymphovascular invasion positive (*n*,%)			35 (32.7%)	14 (16.1%)	0.008 *
**FIGO Tumoral Stage**					>0.05
		IA (*n*,%)	64 (59.8%)	67 (77%)	
		IB (*n*,%)	24 (22.4%)	12 (13.8%)	
		II (*n*,%)	3 (2.8%)	1 (1.1%)	
		IIIA (*n*,%)	2 (1.9%)	1 (1.1%)	
		IIIB (*n*,%)	1 (0.9%)	0	
		IIIC1 (*n*,%)	10 (9.3%)	3 (3.4%)	
		IIIC2 (*n*,%)	3 (2.8%)	2 (2.3%)	
		IVB (*n*,%)	0	1 (1.1%)	

**Table 3:** Follow-up and adjuvant treatment (*n* = total number of patients; SD = standard deviation; ICG = indocyanine green; Tc99m = Technetium99; BT = brachytherapy; RT = external radiotherapy; QT = chemotherapy; * significant differences).

Characteristics		Group 1 (Tc99m + ICG) *n* = 107	Group 2 (ICG) *n* = 87	*p* Value
**ESMO Risk Groups** [30]				>0.05
	Low	44 (41.1%)	54 (6%)	
	Intermediate	27 (25.2%)	14 (16.1%)	
	High-intermediate	25 (23.4%)	12 (13.6%)	
	High	11 (10.3%)	7 (8%)	
**Adjuvant Treatment**				>0.05
	Follow up/BT (*n*,%)	75 (70.1%)	75 (86.2%)	
	RT + BT (*n*,%)	8 (7.5%)	2 (2.3%)	
	QT + RT/QT + RT + BT/QT + BT/QT (*n*,%)	24 (22.4%)	10 (11.5%)	
**Follow-up Time in Days (mean, SD)**		1504 ± 593	423 ± 298	<0.001 *
**Disease-free survival in days (median, range)**		1434 (988–1896)	339 (158–648)	<0.001 *
**Overall survival in days (median, range)**		1510 (1155–1896)	400 (158–694)	<0.001 *
**Relapse (*n*,%)**		21 (19.6%)	6 (6.9%)	0.012 *
	Local Relapse (*n*,%)	5 (4.7%)	3 (3.4%)	
	Metastatic Relapse (*n*,%)	13 (12.1%)	1 (1.2%)	
	Local & Metastatic Relapse (*n*,%)	3 (2.8%)	2 (2.3%)	
**Total Deaths (*n*,%)**		14 (14.3%)	1 (1.2%)	0.001 *
	Oncologic Deaths (*n*,%)	10 (9.3%)	0 (0%)	>0.05

## Data Availability

The data presented in this study are available on request from the corresponding authors. The availability of the data is restricted to investigators based in academic institutions.
